# Peppermint Oil Decreases the Production of Virulence-Associated Exoproteins by *Staphylococcus aureus*

**DOI:** 10.3390/molecules16021642

**Published:** 2011-02-15

**Authors:** Jing Li, Jing Dong, Jia-Zhang Qiu, Jian-Feng Wang, Ming-Jing Luo, Hong-En Li, Bing-Feng Leng, Wen-Zhi Ren, Xu-Ming Deng

**Affiliations:** Key Laboratory of Zoonosis, Ministry of Education, Institute of Zoonosis, College of Animal Science and Veterinary Medicine, Jilin University, Changchun 130062, China; E-Mails: lijing5221@126.com (J.L.); dongjingletter@163.com (J.D.); qiujiazhang1983@163.com (J.-Z.Q.); wjf927@126.com (J.-F.W.); luomingjing@gmail.com (M.-J.L.); empyreal614@gmail.com (H.-E.L.); llcffnn@gmail.com (B.-F.L.)

**Keywords:** *Staphylococcus aureus*, peppermint oil, α-toxin, staphylococcal enterotoxins, toxic shock syndrome toxin 1

## Abstract

The present study aimed to evaluate the antimicrobial activity of peppermint oil against *Staphylococcus aureus*, and further investigate the influence of peppermint oil on *S. aureus* virulence-related exoprotein production. The data show that peppermint oil, which contained high contents of menthone, isomenthone, neomenthol, menthol, and menthyl acetate, was active against *S. aureus* with minimal inhibitory concentrations (MICs) ranging from 64-256 µg/mL, and the production of *S. aureus* exotoxins was decreased by subinhibitory concentrations of peppermint oil in a dose-dependent manner. The findings suggest that peppermint oil may potentially be used to aid in the treatment of *S. aureus* infections.

## 1. Introduction

*Staphylococcus aureus* is a ubiquitous bacterium that can cause minor skin and soft-tissue infections. However, *S. aureus* is also associated with diseases that have high morbidity and mortality such as toxic shock syndrome (TSS) and necrotizing pneumonia [1]. Due to the rapid emergence of antibiotic resistance, and the existence of strains resistant to virtually all antibiotics of clinical value [2], *S. aureus* infections are becoming increasingly difficult to treat, and new therapeutics for both the prevention and treatment of these infections need to be urgently developed.

*S. aureus* also produces a large number of secreted virulence factors that contribute to colonization and lesion progression. These secreted virulence factors are primarily expressed during post-exponential and stationary phase [3] and include a large group of exoenzymes such as glycerol ester hydrolase (lipase) and proteases. A large group of exotoxins are also secreted by *S. aureus*, including highly inflammatory cytolysins (mainly α, β, γ, and δ toxins) and superantigens (SAgs) such as enterotoxins (SEs) and toxic shock syndrome toxin-1 (TSST-1).

α-Toxin is a well-characterized hemolytic toxin that is highly conserved between *S. aureus* strains. This virulence factor possesses pore-forming activity towards a variety of host cells, including erythrocytes, monocytes, and lymphocytes, and is lethal in animal models of *S. aureus* bacteremia [2]. SEs are proteins between 27-30 kDa [4] that cause staphylococcal gastroenteritis and food poisoning in humans [5]. TSST-1, secreted by some *S. aureus* strains, is the key virulence factor that contributes to toxic shock syndrome (TSS), a potentially life-threatening staphylococcal infection with acute onset [6]. Furthermore, both SEs and TSST-1 can function as superantigens, and their abilities to stimulate T-cell proliferation and the release of T-cell-derived cytokines (e.g*.*, TNF-α) have been characterized extensively [7].

Peppermint oil (*Menthae piperitae aetheroleum*) is a naturally occurring carminative that is typically obtained from the fresh leaves of peppermint (*Mentha piperita* L.) by steam distillation [8,9]. It has been used to treat recurrent abdominal pain, irritable bowel syndrome (IBS), nausea, and coughs and colds [10]. Recent studies have also indicated that peppermint oil possesses antibacterial and antifungal activities [11,12]. In this study, we evaluated the anti-*S. aureus* activity of peppermint oil and investigated the effect of subinhibitory concentrations of peppermint oil on virulence-related exoprotein expression by both methicillin-sensitive *S. aureus* (MSSA) and methicillin-resistant *S. aureus* (MRSA) strains.

## 2. Results and Discusssion

### 2.1. Chemical composition of peppermint oil

Peppermint oil was extracted by water distillation and analysed by GC/MS. The chemical composition of the peppermint oil is shown in Table 1. 

### 2.2. Effects of peppermint oil on S. aureus growth

The minimum inhibitory concentration (MIC) of peppermint oil against 28 clinical *S. aureus* isolates ranged between 64 to 256 µg/mL (Table 2). The MIC value of peppermint oil against *S. aureus* strains ATCC 29213, MRSA 2985 and MRSA 3701 was 128 µg/mL. These data suggest that peppermint oil may warrant further investigation for its potential therapeutic efficacy in *S. aureus* infections.

**Figure 1:**
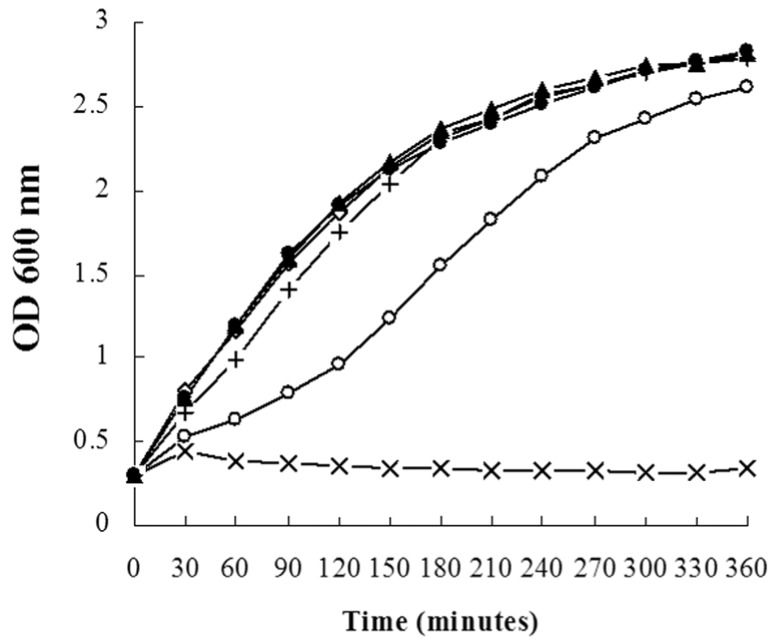
Growth curves for *S. aureus* ATCC 29213 cultured in MHB with different concentrations of peppermint oil. (◇) represents untreated *S. aureus*; (●) represents *S. aureus* cultured with 8 µg/mL peppermint oil; (▲) represents *S. aureus* cultured with 16 µg/mL peppermint oil; (+) represents *S. aureus* cultured with 32 µg/mL peppermint oil; (○) represents *S. aureus* cultured with 64 µg/mL peppermint oil; and (×) represents *S. aureus* cultured with 128 µg/mL peppermint oil.

Growth curve assays were performed to monitor the effect of different peppermint oil concentrations on *S. aureus* growth. As shown in Figure 1, peppermint oil at 1/16 × MIC (8 µg/mL), 1/8 × MIC (16 µg/mL) and 1/4 × MIC (32 µg/mL) did not significantly affect *S. aureus* growth. However, when cultured in 1/2 × MIC (64 µg/mL) of peppermint oil, the *S. aureus* growth rate was significantly decreased; after 30, 120 and 240 min of peppermint oil treatment, the OD_600_ values were 60.7%, 51.6% and 81.3% of the control culture, respectively. When treated with the 1 × MIC (128 µg/mL) of peppermint oil, the growth of *S. aureus* was completely inhibited. Although growth kinetics can vary greatly between bacterial strains, the growth of MRSA 2985 and MRSA 3701 were affected similarly by these concentrations of peppermint oil; cultures supplemented with 1/16, 1/8, and 1/4 of the MIC of peppermint oil had no significant affect on MRSA 2985 and MRSA 3701 growth (data not shown).

### 2.3. Peppermint oil decreases α-toxin, TSST-1, SEA and SEB levels in S. aureus culture supernatants

**Figure 2:**
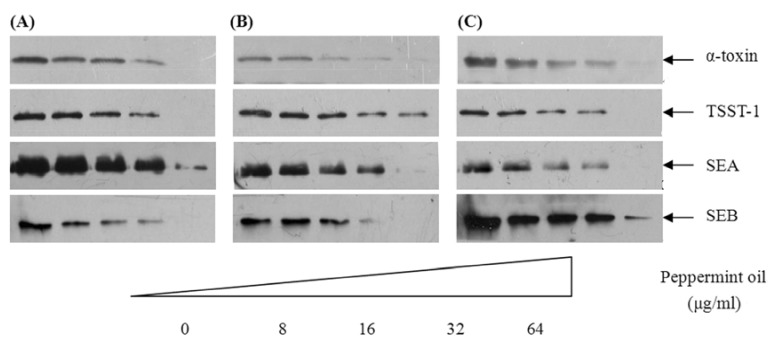
Western blot analysis of α-toxin, TSST-1, SEA and SEB production. *S. aureus* strains ATCC 29213 (A), MRSA 2985 (B) and MRSA 3701 (C) were cultured to the post-exponential growth phase in the absence or presence of increasing concentrations of peppermint oil, the culture supernatants were collected and then subjected to Western blot analysis.

We used Western blot analysis to determine the levels of α-toxin, TSST-1, SEA and SEB produced by *S. aureus*. Because the majority of extracellular proteins are secreted during the post-exponential growth phase, *S. aureus* strains ATCC 29213, MRSA 2985 and MRSA 3701 were cultured in the presence of increasing concentrations of peppermint oil (8 to 64 µg/mL) to an OD_600_ of 2.5 based on the growth curves. As shown in Figure 2, peppermint oil decreases the production of α-toxin, TSST-1, SEA and SEB in a dose-dependent fashion. Incubating the bacteria in 1/16 × MIC of peppermint oil resulted in a marked reduction of α-toxin, TSST-1, SEA and SEB secretion. At 1/2 × MIC, the supernatants from strains ATCC 29213, MRSA 2985 and MRSA 3701 contained little or no immunoreactive proteins.

### 2.4. Peppermint oil inhibits hemolytic activities in S. aureus culture supernatants

α-Toxin is the major secreted protein responsible for the hemolytic activity of *S. aureus*. Therefore, a hemolysin assay was performed to assess whether the reduction in α-toxin observed upon treatment with peppermint oil was biologically relevant. As shown in Table 3, treating *S. aureus* strains ATCC 29213, MRSA 2985 and MRSA 3701 with 1/16 × MIC of peppermint oil reduced hemolytic activity in the culture supernatants to 76.4%, 81.5% and 70.4% of the control, respectively. Remarkably, no hemolytic activity was detected in the supernatants of bacteria cultured in 1/2 × MIC of peppermint oil. Peppermint oil decreased hemolytic activity in a dose-dependent manner in all strains tested.

### 2.5. Peppermint oil inhibits the transcription of hla, tst, sea, seb, and agrA by S. aureus

**Figure 3:**
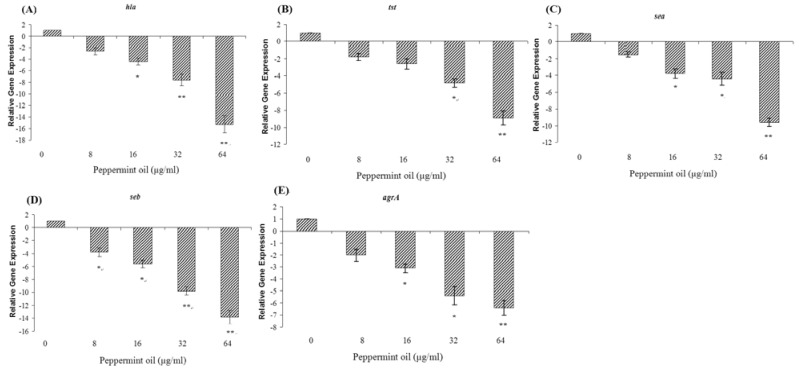
Relative expression levels of *hla* (A), *tst* (B), *sea* (C), *seb* (D), and *agrA* (E) in *S. aureus*. *S. aureus* ATCC 29213 were cultured with different subinhibitory concentrations of peppermint oil to the post-exponential growth phase. Expression levels of *hla*, *tst*, *sea*, *seb*, and *agrA* were monitored by quantitative RT-PCR. Expression was normalized to drug-free cultures. Error bars (n = 3) represent standard deviation,* indicates *p* < 0.05, and ** indicates *p* < 0.01.

Because Western blot analysis revealed that peppermint oil decreases *S. aureus* expression of α-toxin, TSST-1, SEA and SEB in a dose-dependent fashion, we performed a quantitative RT-PCR assay to determine whether peppermint oil affects the transcription of *hla, tst, sea* and *seb*. Since all of these genes are positively controlled by the Agr two-component system [13], we measured *agrA* transcript levels. As expected, peppermint oil significantly repressed the transcription of these genes by *S. aureus* strain ATCC 29213 in a dose-dependent fashion (Figure 3). Treatment with 1/2 × MIC of peppermint oil decreased *hla, tst, sea, seb,* and *agrA* transcription levels by 15.3-, 8.9-, 9.6-, 13.8-, and 6.4-fold, respectively.

### 2.6. Discussion

The worldwide prevalence of methicillin-resistant *S. aureus* and the rapid emergence of vancomycin-intermediate and vancomycin-resistant *S. aureus* strains (VRSA) have not only had a major impact on how antibiotics are used, but have also motivated the pharmaceutical and food industries to develop novel antimicrobial agents [14]. Numerous studies to date have focused on plant extracts, particularly essential oils, for their potent antimicrobial properties against many microorganisms [15]. In this study, peppermint oil was extracted by water distillation and analysed by GC/MS. The data show that peppermint oil, which contained high contents of menthone, isomenthone, neomenthol, menthol, and menthyl acetate, inhibits the growth of both MSSA and MRSA. However, It is often quite difficult to compare the results obtained from different studies, because the compositions of the essential oils can vary greatly depending upon the geographical region, the variety, the age of the plant, the method of drying and the method of extraction of the oil.

The emergence of antibiotic-resistant microorganisms has necessitated alternative therapeutic strategies, and anti-virulence therapy is currently of great interest [16]. The pathogenicity of *S. aureus*, to a great extent, depends upon the secretion of numerous virulence factors. Therefore, the clinical value of antimicrobial agents that treat *S. aureus* infections is determined not only by their respective bactericidal or bacteriostatic activity, but also by their effects on virulence factor secretion [17]. Anti-virulence therapy aims to silence bacterial virulence gene expression, providing opportunities to attenuate pathogenicity and its consequences without placing immediate life-or-death pressure on the target pathogens [18].

Many antibiotics inhibit bacterial virulence factor expression, even at subinhibitory concentrations that have little or no influence on bacterial growth. For instance, subinhibitory concentrations of clindamycin, linezolid, and quinupristin/dalfopristin attenuate the expression of *S. aureus* virulence-associated exoproteins such as α-toxin, SEA, and SEB [17,19,20] and these antibiotics are recommended to treat severe staphylococcal diseases caused by these toxins. Furthermore, it has also been shown that some plant essential oils (e.g., oils of cinnamon, bay and clove) can affect production of exotoxins when used at subinhibitory concentrations, indicating their considerable importance in the food and pharmaceutical industries and an exciting area for further development [15]. In this study, we used transcriptional, expression and phenotypic analyses to show that sublethal concentrations of peppermint oil reduce α-toxin, SEA and SEB, and TSST-1 expression in both MSSA and MRSA in a dose-dependant fashion. 

Furthermore, these findings may be of great significance to the food industry. Staphylococcal food poisoning does not result from the ingestion of *S. aureus* but rather from the ingestion of one or more preformed staphylococcal enterotoxins in *S. aureus*-contaminated food [21]. SEs are heat stable and resistant to strong acids and alkaline solutions. SEA is the most potent toxin of the SEs; less than 200 ng of SEA causes food-borne illness in humans, and its toxicity persists after a 30 min exposure to 100 °C [4]. Because of the negative stigma associated with chemical preservatives such as NaCl and nitrates, natural antimicrobial substances from animal and plant sources are very desirable [22]. Peppermint oil has been used extensively as both a medically relevant compound and as a flavoring agent in foods and confectionery [23]. The U.S. Food and Drug Administration lists peppermint oil as being “generally recognized as safe” [23]. Because peppermint oil inhibits the growth of *S. aureus* and suppresses the expression of SEs, it may be useful as an antimicrobial food additive. However, it should be taken into consideration that the results reported here were achieved after culture in MHB. Experiments are needed to establish if a similar reduction can be achieved in food, especially as the structure and properties of the food matrix are likely to influence toxin production.

Genes encoding virulence factors are coordinately regulated by several intracellular and extracellular signals such as *agr*, *sar*, and *sae* [24]. Subinhibitory concentrations of antibiotics have been shown to interfere with the translation of one or more *S. aureus* regulatory gene products, thereby affecting the transcription of exoprotein-encoding genes [19,30]. Similarly, we wanted to test the hypothesis that the peppermint oil-mediated inhibition of global regulators might reduce the secretion of virulence-related exoproteins. Using quantitative RT-PCR, we found that peppermint oil significantly decreases *agrA* transcription. Because the expression levels of α-toxin, TSST-1, SEA and SEB are positively controlled by *agr* [13,25], subinhibitory concentrations of peppermint oil may reduce the production of these toxins by inhibiting the *agr* two-component system. Nevertheless, the mechanisms by which *S. aureus* regulates virulence gene expression are extremely intricate and involve a hierarchical regulatory cascade of *agr*, *sar*, and other regulatory gene products [26]. Thus, peppermint oil may also affect toxin expression via other mechanisms.

## 3. Experimental

### 3.1. Bacterial strains and reagents

MSSA strain ATCC 29213 was obtained from the American Type Culture Collection (ATCC, Manassas, VA). Twenty-eight *S. aureus* isolates (8 MSSA and 20 MRSA) were acquired from clinical samples from the First Hospital of Jilin University; the clinical MRSA 2985 and 3701 strains, which have the ability to produce α-toxin, TSST-1, SEA and SEB, were chosen for further experiments. Mueller–Hinton broth (MHB) was purchased from BD Biosciences, Inc. (Sparks, MD, USA). Oxacillin and gentamicin were obtained from the National Institute for the Control of Pharmaceutical and Biological Products (Beijing, China), Stock solutions of peppermint oil were prepared in dimethylsulfoxide (DMSO) (Sigma-Aldrich, St. Louis, MO, USA).

### 3.2. Plant material and essential oil extraction

Peppermint (*Mentha piperita* L) was collected in Jianxi Province of China in May 2009. The plant material was air dried in a shady and aerated room until the weight was stable. Peppermint oil was isolated by water distillation for 3 h from the air dried material, using a Clevenger-type apparatus.

### 3.3. Gas chromatography/mass spectral analysis

The chemical composition of the peppermint oil was determined by gas chromatography coupled to mass spectroscopy performed on a GCMS-QP2010 Plus Gas Chromatograph/Mass Spectrometer (Shimadzu Co., Ltd., Kyoto, Japan) equipped with a fused silica capillary column (Rix-5ms; 30 m × 0.25 mm × 0.25μm; Shimadzu, Kyoto, Japan). The carrier gas was helium (1 mL/min). The oven temperature was kept at 60 °C for 10 min and programmed to reach 250 °C at a rate of 10 °C /min. The split ratio was adjusted to 100:1. The injector temperature was set at 280 °C. Mass spectra were obtained by electronic impact at 70 V. Mass range was from *m/z* 50 to 500. The identification of the constituents was performed by a computer-based library search.

### 3.4. MIC determination

MICs (Minimal Inhibitory Concentrations) of peppermint oil for *S. aureus* were assessed in triplicate by a broth microdilution method [27,28]. All tests were performed in MHB supplemented with Tween-80 (Sigma-Aldrich) at a final concentration of 0.5% (v/v). The MICs were defined as the lowest drug concentration at which no visible growth was observed. Oxacillin and gentamycin served as positive controls. 

### 3.5. Growth curves

*S. aureus* strain ATCC 29213 was cultured in MHB supplemented with 0.5% Tween-80 at 37 °C, shaken at 200 rpm to an optical density (OD_600_) of 0.3, and aliquoted into six 250-mL Erlenmeyer flasks in a volume of 100 mL. Five cultures were supplemented with peppermint oil that had been dissolved in DMSO to obtain final concentrations of 1/16 × MIC, 1/8 × MIC, 1/4 × MIC, 1/2 × MIC and 1 × MIC. The final DMSO concentration for all cultures was 1% (v/v). The control culture contained 1% DMSO alone. After adding peppermint oil (or DMSO), the bacteria were grown at 37 °C under aerobic conditions and constant shaking, and cell growth was monitored by reading the OD_600_ values every 30 min.

### 3.6. Growth conditions

For Western blot and hemolysin analysis, overnight cultures of MSSA ATCC 29213, MRSA 2985, and MRSA 3701 were grown in MHB and diluted 30-fold into 500 mL of pre-warmed MHB supplemented with 0.5% Tween-80. After incubation at 37 °C for 30 min under aerobic conditions, cultures were divided into 100 mL aliquots. Increasing concentrations of peppermint oil (1/16, 1/8, 1/4, and 1/2 of the MIC) were added to the bacterial suspensions and incubated to the post-exponential growth phase (OD_600_ of 2.5, equivalent to 1.0 × 10^9^ colony forming units/mL) with constant shaking. Control cultures were treated with 1% DMSO. For the quantitative RT-PCR assay, strain ATCC 29213 was grown to post-exponential growth phase (240 min) in MHB supplemented with 0.5% Tween-80 at 37 °C with increasing concentrations of peppermint oil or 1% DMSO.

### 3.7. Western blot analysis

Bacterial samples described above were centrifuged (5,500 × g at 4 °C for 1 min), the supernatant was removed, and any residual cells were removed with a 0.2 μm filter. Twenty-five µL of culture supernatant was loaded on a 12% sodium dodecyl sulfate polyacrylamide gel after boiling in Laemmli sample buffer [29]. Western blot analysis was performed as previously described [30] and following the manufacturer’s instructions included with the Amersham ECL Western blotting detection reagents (GE Healthcare, Buckinghamshire, UK). Antibodies that recognize α-toxin, SEA and SEB were purchased from Sigma-Aldrich. The anti-TSST-1 antibody was purchased from Santa Cruz Biotechnology (Santa Cruz, CA, USA).

### 3.8. Hemolysin assay

Hemolytic activities of bacterial culture supernatants were assessed using our previously described method [31]. Bacterial samples described above were centrifuged (5,500 × g at 4 °C for 1 min), the supernatant was removed, and any residual cells were removed with a 0.2 μm filter. Before adding defibrinated rabbit blood (25 µL), culture supernatant (0.1 mL) was diluted with PBS buffer (0.9 mL). After incubation for 15 min at 37 °C, unlysed blood cells were pelleted by centrifugation (5,500 × g at room temperature for 1 min). The hemolytic activity of the supernatant was detected by measuring the OD at 543 nm. Hemolytic activity in the control culture was regarded as 100%, and the percent hemolysis was calculated by comparison with the control culture. 

### 3.9. Quantitative RT-PCR

RNA was prepared as described by Xiang *et al*. [32]. Briefly, cells were harvested by centrifugation (5,000 × g for 5 min at 4 °C) and resuspended in TES buffer (10 mM Tris-Cl, 1 mM EDTA, 0.5% SDS) containing 100 µg/mL of lysostaphin (Sigma-Aldrich). Following incubation at 37 °C for 10 min, a Qiagen RNeasy Maxi column was used to isolate total bacterial RNA following the manufacturer’s instructions. Contaminating DNA was removed using the on-column RNase-free DNase I step (Qiagen, Hilden, Germany). RNA concentrations were detected at an A_260_ and loaded onto an RNase-free 2% agarose gel to assess generalized degradation. The primer pairs used in quantitative RT-PCR are listed in Table 4. RNA was reverse transcribed to cDNA using the Takara RNA PCR kit (AMV) Ver. 3.0 (Takara, Kyoto, Japan) according to the manufacturer’s protocol. The resulting cDNA was stored at -20 °C. PCR reactions were performed in a 25 µL volume with SYBR Premix Ex TaqTM (Takara) as recommended by the manufacturer. The reactions were performed using a 7000 Sequence Detection System (Applied Biosystems, Courtaboeuf, France). The cycling conditions were as follows: 95 °C for 30 s; 40 cycles at 95ºC for 5 s, 55 °C for 30 s, and 72 °C for 40 s. We performed one dissociation step at 95 °C for 15 s, 60 °C for 30 s, and 95 °C for 15 s. All samples were analyzed in triplicate, and the *16S rRNA* housekeeping gene served as an internal control to normalize expression levels between samples. Relative expression levels were analyzed using the ΔΔCt method as described in the Applied Biosystems User Bulletin No. 2.

### 3.10. Statistical analysis

The experimental data were analyzed with SPSS 12.0 statistical software and presented as means value ± SD. An independent Student’s t-test was used to determine statistical differences, and a *p* value less than 0.05 was considered to be statistically significant.

## 4. Conclusions

In the present study, we have investigated the antimicrobial activity of peppermint oil against *Staphylococcus aureus*, and further investigated the influence of peppermint oil on *S. aureus* virulence-related exoprotein production. The data indicated that peppermint oil, which contained high contents of menthone, isomenthone, neomenthol, menthol, and menthyl acetate, was active against *S. aureus* with minimal inhibitory concentrations (MICs) ranging from 64-256 µg/mL, and the production of *S. aureus* exotoxins was decreased by subinhibitory concentrations of peppermint oil in a dose-dependent manner.

## Figures and Tables

**Table 1:** The main components of peppermint oil and their relative contents (%).

Compound	Retention time (min)	Relative contents (%)
α-Pinene	4.383	1.72
β-Pinene	5.017	1.17
3-Octanol	5.150	1.5
Limonene	5.717	0.9
Eucalyptol	5.792	0.3
Isopulegol	7.575	1.5
Menthone	7.692	15.98
Isomenthone	7.842	7.89
Neomenthol	7.900	5.96
Menthol	8.017	48.44
*p*-Menth-1-en-8-ol	8.283	0.63
*n*-Valeric acid *cis*-3-hexenyl ester	8.767	0.3
Pulegone	8.958	1.29
2-Cyclohexen-1-one	9.033	1.55
1-Decanol	9.200	0.49
Menthyl acetate	9.642	3.17
γ-Elemene	11.150	0.41
Caryophyllene	11.683	0.44
Caryophyllene oxide	13.900	0.54

**Table 2:** MICs of peppermint oil, oxacillin, and gentamicin against 28 clinical isolates of *S. aureus.*

Organism	Antimicrobial agent	MIC (μg/mL)*^a^*		
		Range	50%	90%
MSSA (8)	Peppermint oil	64-128	64	128
	Oxacillin	0.5-4	2	2
	Gentamycin	0.06-32	2	4
MRSA (20)	Peppermint oil	64-256	128	128
	Oxacillin	16->256	128	256
	Gentamycin	0.125-128	8	32

*^a^* 50% and 90%, MICs at which 50% and 90% of the isolates are inhibited, respectively.

**Table 3:** Hemolytic activities in the supernatant of *S. aureus* grown in increasing concentrations of peppermint oil.

*S. aureus* strain	Hemolysis (%) of rabbit erythrocytes by culture supernatant^a^				
	0	8 µg/mL	16 µg/mL	32 µg/mL	64 µg/mL
ATCC 29213	100%	76.4% ± 5.2	37.3% ± 7.2^*^	24.2% ± 6.5^**^	none^b^
MRSA 2985	100%	81.5% ± 7.2	34.6% ± 5.3^*^	18.6% ± 4.4^**^	none
MRSA 3701	100%	70.4% ± 8.3	25.3% ± 5.8^**^	10.8% ± 3.2^**^	none

*^a^* Hemolytic activity in untreated *S. aureus* culture supernatants was set to 100%; *^b^* none indicates no hemolytic activity was observed; Values represent means ± SD (n = 3). ^*^ indicates *p* < 0.05 and ^**^ indicates *p* < 0.01 compared to the corresponding control.

**Table 4:** Primers used for quantitative RT-PCR.

Primer	Sequence	Genomic Location
*16S rRNA*-fw	5'-GCTGCCCTTTGTATTGTC-3'	287-305
*16S rRNA*-rv	5'-AGATGTTGGGTTAAGTCCC-3'	446-465
*hla* –fw	5'-TTGGTGCAAATGTTTC-3'	485-501
*hla*-rv	5'-TCACTTTCCAGCCTACT-3'	569-586
*sea*-fw	5'-ATGGTGCTTATTATGGTTATC-3'	335-356
*sea*-rv	5'-CGTTTCCAAAGGTACTGTATT-3'	477-498
*seb*-fw	5'-TGTTCGGGTATTTGAAGATGG-3'	480-501
*seb*-rv	5'-CGTTTCATAAGGCGAGTTGTT-3'	612-633
*tst*-fw	5'-ACCCCTGTTCCCTTATCATC-3'	73-93
*tst*-rv	5'-AAAAGCGTCAGACCCACTAC-3'	159-180
*agrA-* fw	5'-TGATAATCCTTATGAGGTGCTT-3'	111-133
*agrA-* rv	5'-CACTGTGACTCGTAACGAAAA-3'	253-274
